# Supplementary material for: Magnetic and Electrical Characteristics of Nd3+-Doped Lead Molybdato-Tungstate Single Crystals
Source: Materials (Basel). 2023 Jan 9;16(2):620. doi: 10.3390/ma16020620 (PMC9866789; doi:10.3390/ma16020620)
Supplement: Supplementary file 1 [file materials-16-00620-s001.zip › materials-2094343-supplementary.docx]

**Supplementary data: Tables S1–S8**

**CIF files for PNMWO single crystals have been deposited in the CCDC database with the following Nos.**

**CSD 2221426-2221427.**

**Table S1.** Crystal data and structure refinement for PNMWO single crystal (*x* = 0.001).

| **Empirical formula** | **Pb_0.997_Nd_0.002_(MoO_4_)_0.997_(WO_4_)_0.003_** |
| --- | --- |
| Formula weight | 367.07 |
| Temperature | 293(1) K |
| Wavelength | 0.71073 Å |
| Crystal system | Tetragonal |
| Space group | I 4_1_/a |
| Unit cell dimensions | a = 5.4380(4) Å α= 90° |
|  | b = 5.4380(4) Å |
|  | c = 12.1111(13) Å α= 90° |
| Volume | 358.15(5) Å^3^ |
| Z | 4 |
| Density (calculated) | 6.809 Mg/m^3^ |
| Absorption coefficient | 50.292 mm^−1^ |
| F(000) | 624 |
| Crystal size | 0.04 × 0.04 × 0.02 mm^3^ |
| Theta range for data collection | 4.11 to 40.47° |
| Index ranges | -7<=h<=9, -9<=k<=8, -21<=l<=20 |
| Reflections collected | 4099 |
| Independent reflections | 558 [R(int) = 0.0331] |
| Completeness to theta = 40.47° | 98.6% |
| Absorption correction | Semi-empirical from equivalents |
| Max. and min. transmission | 1.00000 and 0.32374 |
| Refinement method | Full-matrix least-squares on F^2^ |
| Data / restraints / parameters | 558/0/15 |
| Goodness-of-fit on F^2^ | 0.854 |
| Final R indices [I>2sigma(I)] | R1 = 0.0149, wR2 = 0.0366 |
| R indices (all data) | R1 = 0.0199, wR2 = 0.0410 |
| Extinction coefficient | 0.00228(16) |
| Largest diff. peak and hole | 1.079 and −1.556 (e Å^−3^) |

**Table S2.** Atomic coordinates (×10^4^) and equivalent isotropic displacement parameters (Å^2^ × 10^3^) for PNMWO single crystal (*x* = 0.001). U(eq) is defined as one third of the trace of the orthogonalized Uij tensor.

| **Element** | **x** | **y** | **z** | **U(eq)** |
| --- | --- | --- | --- | --- |
| Pb/Nd | 0 | 2500 | 1250 | 12(1) |
| O | 1372(3) | −148(3) | 2943(1) | 16(1) |
| Mo/W | 0 | 2500 | 6250 | 9(1) |

**Table S3.** Bond lengths [Å] and angles [°] for PNMWO single crystal (*x* = 0.001).

| **Interatomic distances (Å)** | | | | | |
| --- | --- | --- | --- | --- | --- |
| Pb/Nd-O#1 | 2.6145(17) | Pb/Nd-O#5 | 2.6309(16) | Mo/W-O#10 | 1.7739(16) |
| Pb/Nd-O#2 | 2.6145(17) | Pb/Nd-O#6 | 2.6309(16) | Mo/W-O#11 | 1.7739(17) |
| Pb/Nd-O | 2.6145(17) | Pb/Nd-O#7 | 2.6309(16) | Mo/W-O#8 | 1.7739(17) |
| Pb/Nd-O#3 | 2.6145(17) | O-Mo/W#8 | 1.7739(16) |  |  |
| Pb/Nd-O#4 | 2.6309(16) | Mo/W-O#9 | 1.7739(17) |  |  |
| **Angles (°)** | | | | | |
| O#1-Pb/Nd-O#2 | 127.97(5) | O#3-Pb/Nd-O#5 | 78.24(5) | O#5-Pb/Nd-O#7 | 136.40(8) |
| O#1-Pb/Nd-O | 127.97(5) | O#4-Pb/Nd-O#5 | 97.93(3) | O#6-Pb/Nd-O#7 | 97.93(3) |
| O#2-Pb/Nd-O | 76.68(8) | O#1-Pb/Nd-O#6 | 73.86(3) | Mo/W#8-O-Pb/Nd | 135.17(8) |
| O#1-Pb/Nd-O#3 | 76.68(8) | O#2-Pb/Nd-O#6 | 78.24(5) | Mo/W#8-O-Pb/Nd#4 | 120.36(8) |
| O#2-Pb/Nd-O#3 | 127.97(5) | O-Pb/Nd-O#6 | 67.74(4) | Pb/Nd-O-Pb/Nd#4 | 101.76(5) |
| O-Pb/Nd-O#3 | 127.97(5) | O#3-Pb/Nd-O#6 | 149.38(7) | O#9-Mo/W-O#10 | 107.66(5) |
| O#1-Pb/Nd-O#4 | 149.38(7) | O#4-Pb/Nd-O#6 | 136.40(8) | O#9-Mo/W-O#11 | 107.66(5) |
| O#2-Pb/Nd-O#4 | 67.74(4) | O#5-Pb/Nd-O#6 | 97.93(3) | O#10-Mo/W-O#11 | 113.16(11) |
| O-Pb/Nd-O#4 | 78.24(5) | O#1-Pb/Nd-O#7 | 78.24(5) | O#9-Mo/W-O#8 | 113.16(11) |
| O#3-Pb/Nd-O#4 | 73.86(3) | O#2-Pb/Nd-O#7 | 149.38(7) | O#10-Mo/W-O#8 | 107.66(5) |
| O#1-Pb/Nd-O#5 | 67.74(4) | O-Pb/Nd-O#7 | 73.86(3) | O#11-Mo/W-O#8 | 107.66(5) |
| O#2-Pb/Nd-O#5 | 73.86(3) | O#3-Pb/Nd-O#7 | 67.74(4) |  |  |
| O-Pb/Nd-O#5 | 149.38(7) | O#4-Pb/Nd-O#7 | 97.93(3) |  |  |

Symmetry transformations used to generate equivalent atoms:

#1 y-1/4,-x+1/4,-z+1/4 #2 -x+0,-y+1/2,z+0 #3 -y+1/4,x+1/4,-z+1/4

#4 -x+1/2,-y+1/2,-z+1/2 #5 y-1/4,-x+3/4,z-1/4

#6 x-1/2,y,-z+1/2 #7 -y+1/4,x-1/4,z-1/4 #8 -x,-y,-z+1

#9 x,y+1/2,-z+1 #10 -y-1/4,x+1/4,z+1/4 #11 y+1/4,-x+1/4,z+1/4

**Table S4.** Anisotropic displacement parameters (Å^2^ × 10^3^) for PNMWO single crystal (*x* = 0.001). The anisotropic displacement factor exponent takes the form: -2π^2^[h^2^a*^2^U^11^ + ... + 2 h k a* b* U^12^].

| **Element** | **U^11^** | **U^22^** | **U^33^** | **U^23^** | **U^13^** | **U^12^** |
| --- | --- | --- | --- | --- | --- | --- |
| Pb/Nd | 13(1) | 13(1) | 12(1) | 0 | 0 | 0 |
| O | 14(1) | 19(1) | 15(1) | 4(1) | −1(1) | −3(1) |
| Mo/W | 8(1) | 8(1) | 10(1) | 0 | 0 | 0 |

**Table S5.** Crystal data and structure refinement for PNMWO single crystal (*x* = 0.005).

| **Empirical Formula** | **Pb_0.985_Nd_0.010_ (MoO_4_)_0.985_(WO_4_)_0.015_** |
| --- | --- |
| Formula weight | 366.79 |
| Temperature | 293(1) K |
| Wavelength | 0.71073 Å |
| Crystal system | Tetragonal |
| Space group | I 4_1_/a |
| Unit cell dimensions | a = 5.4357(4) Å α= 90° |
|  | b = 5.4357(4)Å β= 90° |
|  | c = 12.1067(14) Å γ= 90° |
| Volume | 357.71(6) Å^3^ |
| Z | 4 |
| Density (calculated) | 6.817 Mg/m^3^ |
| Absorption coefficient | 50.353 mm^−1^ |
| F(000) | 624 |
| Crystal size | 0.04 × 0.04 × 0.04 mm^3^ |
| Theta range for data collection | 4.11 to 40.59° |
| Index ranges | -9<=h<=8, -9<=k<=9, -16<=l<=22 |
| Reflections collected | 4843 |
| Independent reflections | 559 [R(int) = 0.0392] |
| Completeness to theta = 40.59° | 97.2% |
| Absorption correction | Semi-empirical from equivalents |
| Max. and min. transmission | 1.00000 and 0.14732 |
| Refinement method | Full-matrix least-squares on F^2^ |
| Data / restraints / parameters | 559/0/15 |
| Goodness-of-fit on F^2^ | 1.036 |
| Final R indices [I>2sigma(I)] | R1 = 0.0164, wR2 = 0.0388 |
| R indices (all data) | R1 = 0.0188, wR2 = 0.0405 |
| Extinction coefficient | 0.0087(3) |
| Largest diff. peak and hole | 1.777 and −1.370 (e Å^−3^) |

**Table S6.** Atomic coordinates (×10^4^) and equivalent isotropic displacement parameters (Å^2^ × 10^3^) for PNMWO single crystal (*x* = 0.005).

| **Element** | **x** | **y** | **z** | **U(eq)** |
| --- | --- | --- | --- | --- |
| Pb/Nd | 0 | 2500 | 1250 | 12(1) |
| O | 1370(3) | −148(3) | 2941(1) | 16(1) |
| Mo/W | 0 | 2500 | 6250 | 9(1) |

U(eq) is defined as one third of the trace of the orthogonalized Uij tensor.

**Table S7.** Bond lengths [Å] and angles [°] for PNMWO single crystal (*x* = 0.005).

| **Interatomic Distances (Å)** | | | | | | |
| --- | --- | --- | --- | --- | --- | --- |
| Pb/Nb-O#1 | 2.6108(14) | Pb/Nb -O#5 | 2.6313(15) | Mo/W-O#8 | 1.7747(14) | |
| Pb/Nb -O#2 | 2.6108(14) | Pb/Nb -O#6 | 2.6313(15) | Mo/W-O#9 | 1.7747(14) | |
| P/Nb b-O#3 | 2.6108(14) | Pb/Nb -O#7 | 2.6313(15) | Mo/W-O#10 | 1.7747(14) | |
| Pb/Nb -O | 2.6108(14) | O-Mo/W#8 | 1.7747(14) | Mo/W-O#11 | 1.7747(14) | |
| Pb/Nb -O#4 | 2.6313(15) | O-Pb/Nb #4 | 2.6313(15) |  |  | |
| **Angles (°)** | | | | | | |
| O#1-Pb/Nb -O#2 | 76.73(7) | O-Pb/Nb -O#5 | 67.70(3) | O#5-Pb/Nb -O#7 | | 97.97(2) |
| O#1-Pb/Nb -O#3 | 127.93(4) | O#4-Pb/Nb -O#5 | 136.28(6) | O#6-Pb/Nb -O#7 | | 136.28(6) |
| O#2-Pb/Nb -O#3 | 127.93(4) | O#1-Pb/Nb -O#6 | 78.21(5) | Mo/W#8-O-Pb/Nb | | 135.26(7) |
| O#1-Pb/Nb -O | 127.93(4) | O#2-Pb/Nb -O#6 | 67.70(3) | Mo/W#8-O-P/Nb b#4 | | 120.21(7) |
| O#2-Pb/Nb -O | 127.93(4) | O#3-Pb/Nb -O#6 | 149.46(5) | Pb/Nb -O-Pb/Nb #4 | | 101.79(5) |
| O#3-Pb/Nb -O | 76.73(7) | O-Pb/Nb -O#6 | 73.89(3) | O#8-Mo/W-O#9 | | 112.99(9) |
| O#1-Pb/Nb -O#4 | 149.46(5) | O#4-Pb/Nb -O#6 | 97.97(2) | O#8-Mo/W-O#10 | | 107.74(5) |
| O#2-Pb/Nb -O#4 | 73.89(3) | O#5-P/Nb b-O#6 | 97.97(2) | O#9-Mo/W-O#10 | | 107.74(5) |
| O#3-P/Nb b-O#4 | 67.70(3) | O#1-Pb/Nb -O#7 | 67.70(3) | O#8-Mo/W-O#11 | | 107.74(5) |
| O-Pb/Nb -O#4 | 78.21(5) | O#2-Pb/Nb -O#7 | 78.21(5) | O#9-Mo/W-O#11 | | 107.74(5) |
| O#1-Pb/Nb -O#5 | 73.89(3) | O#3-Pb/Nb -O#7 | 73.89(3) | O#10-Mo/W-O#11 | | 112.99(9) |
| O#2-Pb/Nb -O#5 | 149.46(5) | O-Pb/Nb -O#7 | 149.46(5) |  | |  |
| O#3-Pb/Nb -O#5 | 78.21(5) | O#4-Pb/Nb -O#7 | 97.97(2) |  | |  |

Symmetry transformations used to generate equivalent atoms:

#1 y-1/4,-x+1/4,-z+1/4 #2 -y+1/4,x+1/4,-z+1/4

#3 -x+0,-y+1/2,z+0 #4 -x+1/2,-y+1/2,-z+1/2 #5 x-1/2,y,-z+1/2

#6 -y+1/4,x-1/4,z-1/4 #7 y-1/4,-x+3/4,z-1/4

#8 -x,-y,-z+1 #9 x,y+1/2,-z+1 #10 -y-1/4,x+1/4,z+1/4

#11 y+1/4,-x+1/4,z+1/4

**Table S8.** Anisotropic displacement parameters (Å^2^×10^3^) for PNMWO single crystal (*x* = 0.005). The anisotropic displacement factor exponent takes the form: -2π^2^[h^2^a*^2^U^11^ + ... + 2 h k a* b*⋅U^12^].

| **Element** | **U^11^** | **U^22^** | **U^33^** | **U^23^** | **U^13^** | **U^12^** |
| --- | --- | --- | --- | --- | --- | --- |
| Pb/Nd | 12(1) | 12(1) | 13(1) | 0 | 0 | 0 |
| O | 16(1) | 17(1) | 16(1) | 4(1) | −1(1) | −2(1) |
| Mo/W | 8(1) | 8(1) | 11(1) | 0 | 0 | 0 |
